# Quantitative proteomic analysis of aqueous humor from patients with drusen and reticular pseudodrusen in age-related macular degeneration

**DOI:** 10.1186/s12886-018-0941-9

**Published:** 2018-11-07

**Authors:** Je-Hyun Baek, Daehan Lim, Kyu Hyung Park, Jae-Byoung Chae, Hyoik Jang, Jonghyun Lee, Hyewon Chung

**Affiliations:** 1 R&D Center for Clinical Mass Spectrometry, Seegene Medical Foundation, Seoul, 04805 South Korea; 2 Department of Ophthalmology, Konkuk University School of Medicine, Konkuk University Medical Center, 120-1 Neungdong-ro, Gwangjin-gu, Seoul, Republic of Korea; 3 Department of Ophthalmology, Seoul National University College of Medicine, Seoul National University Bundang Hospital, Seongnam, 13620 South Korea; 4 Department of Ophthalmology, Ilsan Paik Hospital, Inje University College of Medicine, Goyang, 10380 South Korea

**Keywords:** Age-related macular degeneration, Complement, Drusen, Reticular pseudodrusen, SWATH-MS

## Abstract

**Background:**

To identify novel biomarkers related to the pathogenesis of dry age-related macular degeneration (AMD), we adopted a human retinal pigment epithelial (RPE) cell culture model that mimics some features of dry AMD including the accumulation of intra- and sub-RPE deposits. Then, we investigated the aqueous humor (AH) proteome using a data-independent acquisition method (sequential window acquisition of all theoretical fragment ion mass spectrometry) for dry AMD patients and controls.

**Methods:**

After uniformly pigmented polarized monolayers of human fetal primary RPE (hfRPE) cells were established, the cells were exposed to 4-hydroxy-2-nonenal (4-HNE), followed by Western blotting, immunofluorescence analysis and ELISA of cells or conditioned media for several proteins of interest. Data-dependent acquisition for identification of the AH proteome and SWATH-based mass spectrometry were performed for 11 dry AMD patients according to their phenotypes (including soft drusen and reticular pseudodrusen [RPD]) and 2 controls (3 groups).

**Results:**

Increased intra- and sub-RPE deposits were observed in 4-HNE-treated hfRPE cells compared with control cultures based on APOA1, cathepsin D, and clusterin immunoreactivity. Additionally, the differential abundance of proteins in apical and basal chambers with or without 4-HNE treatment confirmed the polarized secretion of proteins from hfRPE cells. A total of 119 proteins were quantified in dry AMD patients and controls by SWATH-MS. Sixty-five proteins exhibited significantly altered abundance among the three groups. A two-dimensional principal component analysis plot was generated to identify typical proteins related to the pathogenesis of dry AMD. Among the identified proteins, eight proteins, including APOA1, CFHR2, and CLUS, were previously considered major components or regulators of drusen. Three proteins (SERPINA4, LUM, and KERA proteins) have not been previously described as components of drusen or as being related to dry AMD. Interestingly, the LUM and KERA proteins, which are related to extracellular matrix organization, were upregulated in both RPD and soft drusen.

**Conclusions:**

Differential protein expression in the AH between patients with drusen and RPD was quantified using SWATH-MS in the present study. Detailed proteomic analyses of dry AMD patients might provide insights into the in vivo biology of drusen and RPD.

**Electronic supplementary material:**

The online version of this article (10.1186/s12886-018-0941-9) contains supplementary material, which is available to authorized users.

## Background

Age-related macular degeneration (AMD) is the progressive degeneration of the retinal pigment epithelium (RPE), retina, and choriocapillaris observed among elderly people and is one of the leading causes of blindness worldwide [1–3]. AMD is mainly divided into a dry (atrophic) subtype (80–90%) and a wet (neovascular) subtype (10–20%). A hallmark of neovascular AMD is choroidal neovascularization (CNV) with subsequent development of subretinal fluid accumulation, hemorrhage, exudation, and scarring, which can lead to the loss of vision. Currently, vision loss and blindness from neovascular AMD is largely treatable, with the advent of antiangiogenic drugs that mainly target vascular endothelial growth factor (VEGF). With the introduction of anti-VEGF intravitreal injections, the incidence of legal blindness attributable to wet AMD has decreased by 50% in some countries [3, 4].

However, the molecular mechanisms underlying dry AMD remain largely unknown; there is no proven treatment for dry AMD or its progression to geographic atrophy (GA) or for neovascular AMD. Dry AMD is a disease with various phenotypes, such as drusen and reticular pseudodrusen (RPD). Drusen, which represent the major phenotype of AMD, are focal extracellular deposits located between the RPE and Bruch’s membrane (BM) that are associated with the development and progression of AMD [5, 6]. By contrast, RPD, first described in 1990 by Mimoun et al. [7], are located between the RPE and outer retina, as shown by recently evolving imaging techniques and histological studies [5]. Although the pathogenesis of RPD remains unknown, they have been recognized as an additional phenotype of AMD since they have been associated with an increased risk of progression to late forms of AMD, such as neovascular AMD and/or GA [5, 8–10]; therefore, they contribute to loss of vision by inducing atrophy in the outer retina [11].

The occurrence of RPD in Sorsby fundus dystrophy and pseudoxanthoma elasticum has been demonstrated recently [11–13], suggesting that the pathophysiology of RPD might parallel that of these diseases; dysfunction of the choroid-BM-RPE complex might be associated with the development of RPD. In the case of Sorsby fundus dystrophy, mutations in the tissue inhibitor of metalloproteinase-3 (TIMP3) gene could lead to the formation of abnormal deposits between BM and RPE and thus functionally impair the choroid-BM-RPE complex, resulting in atrophy and CNV [12]. Additionally, in the pseudoxanthoma elasticum, damage to BM leads to the development of RPD [11]. The RPE cell is a highly polarized cell type that produces and secretes proteins onto its basolateral and apical surfaces, meeting differential demands on either side of the photoreceptors and choriocapillaris and holding a large majority of the secreted proteins on the basolateral side [14, 15]. Many proteins are preferentially secreted by either the apical or basolateral surface, and their incorrect localization/function may lead to retinal degeneration.

In a previous study, we profiled and characterized the whole proteome of exosomes obtained from the aqueous humor (AH) of patients with neovascular AMD and compared the differential abundance of selected proteins in ARPE-19 cell cultures exposed to oxidative stress [16, 17]. However, neither proteomic analysis of AH from patients with dry AMD nor the pathogenic implications of dry AMD have yet been reported, mainly due to the difficulty in obtaining AH samples from dry AMD patients and the high-tech proteomic methods required to analyze the very small quantities of precious AH samples.

In the present study, to gain insight into the pathogenesis and characteristics of dry AMD and understand the underlying disease mechanisms, we first introduced a primary human fetal RPE (hfRPE) cell polarized culture model and examined the relative abundance of secreted proteins from hfRPE cells exposed to oxidative stress. Then, we investigated the AH proteome of dry AMD patients according to their phenotypes using a data-independent acquisition method (sequential window acquisition of all theoretical fragment ion mass spectrometry [SWATH-MS], a specialized high-resolution mass spectrometric technique providing quantitative accuracy and reproducibility).

## Methods

### Primary hfRPE cell culture and the measurement of transepithelial resistance

Primary hfRPE cells purchased from Lonza Biologics (catalog number: 194987, Lonza, Basel, Switzerland) were grown and maintained in RtEGM™ BulletKit® medium with supplements (catalog numbers: 00195406 and 00195407, respectively, Lonza) at 37 °C under 5% CO_2_. The cells were seeded onto Matrigel (catalog number: 356230, Corning, NY, USA)-precoated cell culture inserts at 4.6 × 10^4^ cells per well (Transwell 0.4-μm pores, polyester membranes; catalog number: 3450, Corning) using 24-mm-diameter inserts. The medium was changed every 2 to 3 days. Only fresh passage 1 cells were used in the experiments, when they exhibited transepithelial resistance (TER) > 500 Ω·cm^2^ and were confluent, with a uniform hexagonal morphology and mature pigmentation, at 6 to 8 weeks after seeding. Confluent monolayers of hfRPE cells with stable TER were treated with 50 or 100 μM 4-hydroxy-2-nonenal (4-HNE) for 24 h to induce oxidative stress.

TER was measured as described previously [18, 19]. Briefly, TER was measured in a cell culture hood immediately within 3 min after the removal of hfRPE cells from the cell incubator when hfRPE cells were confluent with uniform pigmentation, 6 to 8 weeks after seeding. Epithelial Voltohmmeter (EVOM, World Precision Instruments, FL, USA) electrodes were first washed with 70% ethanol and then with PBS. Net TERs were calculated from five independent measurements by subtracting the background resistance values of the blank, Matrigel-coated filter and culture medium from the measured resistance values (Ω·cm^2^) for each treated condition of hfRPE cells.

### Immunofluorescence and Western blotting

For immunofluorescence (IF) assays, monolayers of hfRPE cells cultured on Transwell permeable inserts were fixed with 4% paraformaldehyde (PFA) overnight. Flat mounts of fixed hfRPE cell cultures or 10-μm cryosections were washed three times in PBS and fixed in 4% PFA for 15 min. Next, the samples were permeabilized with PBST (0.1% Triton X-100 in PBS) for 5 min and blocked with blocking solution (5% BSA in PBS) for 1 h at room temperature, followed by incubation overnight at 4 °C with the primary antibodies (Table S1 in Additional file 1). The samples were then washed three times with PBS, incubated for 1 h at room temperature with the secondary antibodies (Table S1 in Additional file 1) and stained with DAPI (1:3000) in PBS for 10 min. Microphotographs were obtained using a fluorescence microscope (Scope A1, Carl Zeiss MicroImaging, Inc., oberkochen, Germany).

For Western blot analysis, 4-HNE-treated hfRPE cells from each set of conditions were lysed in radioimmunoprecipitation assay (RIPA) buffer (Thermo Scientific, MA, USA) containing protease inhibitors (Sigma-Aldrich, MO, USA) and pepstatin A (Sigma). The extracted protein sample was quantitated with a standard bicinchoninic acid (BCA) protein assay. Twenty-microgram protein samples were denatured by adding 1× sample buffer and loaded into the lanes of a 10% Tris-glycine SDS gel. The loaded proteins were subsequently electroblotted onto polyvinylidene difluoride (PVDF) membranes and blocked with 5% skim milk for 1 h at room temperature. Thereafter, the membranes were incubated overnight at 4 °C with primary antibodies (Table S2 in Additional file 1). After overnight incubation, the membranes were incubated for 1 h at room temperature with secondary antibodies (Table S2 in Additional file 1). The membranes were exposed using a luminescent image analyzer (LAS-4000, Fujifilm, Tokyo, Japan). Finally, each membrane was stripped and probed with a loading control antibody.

### ELISA analysis of conditioned media

The conditioned media of hfRPE cells treated with 4-HNE or serum-free media for 24 h were collected. The levels of VEGF-A (catalog number: DVE00; R&D Systems, MN, USA), PEDF (catalog number: PED613; BioProducts MD, LLC, MD, USA), and complement factor H (CFH) (catalog number: HK342–02; Hycultbiotech, Uden, Netherlands) in conditioned media from hfRPE cells were quantitatively assessed using a sandwich ELISA kit. All procedures were performed according to the manufacturer’s protocol. The dilution factors of the conditioned media from hfRPE cells were 20-fold for VEGF-A, 5000-fold for PEDF, and 1-fold for CFH. Color intensities were measured using a microplate reader (Molecular Devices, CA, USA). The standard ranges were 15.6–1000 pg/mL for VEGF-A, 0.03–1.00 ng/mL for PEDF, and 3.9–250 ng/mL for CFH. Triplicate samples were used in all assays. Inter- and intra-assay variation was 4.1% and 6%, respectively.

### Patients and AH sample collection

**Fig. 1:**
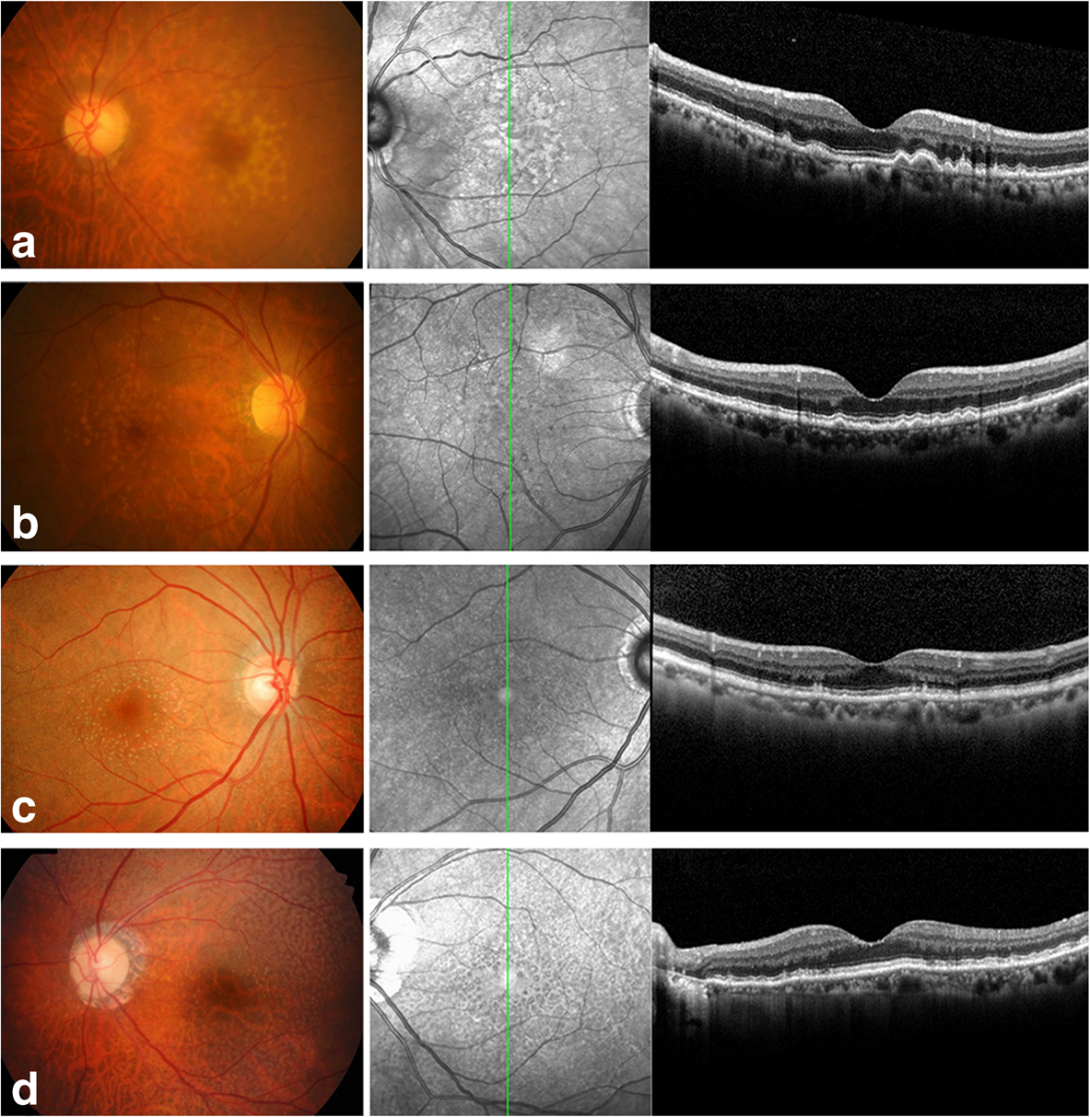
Color fundus photos (left) and optical coherence tomography images (right) from patients with drusen or reticular pseudodrusen (RPD) (patients with dry age-related macular degeneration in Sample Set 2 in Table 1). (**a**) A 71-year-old woman with Drusen, (**b**) an 80-year-old woman with Drusen, (**c**) a 76-year-old woman with RPD, (**d**) a 67-year-old woman with RPD

AH samples were collected at the Department of Ophthalmology, Konkuk University Medical Center, Seoul, Korea, and at the Department of Ophthalmology, Seoul National University Bundang Hospital, Seongnam, Korea. From January 1, 2014, to December 31, 2015, 13 patients undergoing cataract surgery were enrolled in this study. Among these individuals, 11 patients with dry AMD exhibited either drusen or RPD without GA. Two patients presented no retinal diseases, including dry AMD. Patients with other ophthalmic diseases (e.g., glaucoma, uveitis, or progressive retinal disease) or uncontrolled systemic diseases (e.g., uncontrolled diabetes mellitus), or who had undergone laser or intraocular surgery were excluded. All 13 patients underwent routine senile cataract surgery for visual rehabilitation. The extent of the cataracts in each individual corresponded to the patient’s age. The clinical data from the patients and controls are summarized in Table 1 and Fig. 1.

**Table 1:** Summary of the demographic characteristics of dry age-related macular degeneration patients and control subjects

Property	Sample Set 1: DDA^a^ in AH^b^	Sample Set 2: SWATH-MS^c^ in AH		
	Dry AMD^d^	Dry AMD		Control
		Drusen	RPD^e^	
No. of AH samples	7	2	2	2
Age (mean ± SD, years)	74.6 ± 7.5	73.5 ± 4.9		74.5 ± 1.5
Sex (men:women)	3:4	0:4		0:2
Diabetes mellitus (No.)	2	1		0
Hypertension (No.)	3	1		1

^a^*DDA* data-dependent acquisition, ^b^*AH* aqueous humor, ^c^*SWATH-MS* sequential window acquisition of all theoretical fragment ion mass spectrometry, a specialized high-resolution mass spectrometric technique providing quantitative accuracy and reproducibility, ^d^*AMD* age-related macular degeneration, ^e^*RPD* reticular pseudodrusen

All AH samples were obtained immediately before cataract surgery. The collection of all samples was performed using standard sterile procedures, and AH samples were obtained via anterior chamber paracentesis using a 30-gauge needle. No complications were encountered after paracentesis of the anterior chamber. AH samples (100–150 μl) were placed in safe-lock microcentrifuge tubes (1.5 mL), immediately frozen at − 80 °C and stored until analysis. The study followed the guidelines of the Declaration of Helsinki, and informed written consent was obtained from all patients and control subjects. The procedure for AH collection was approved by the Institutional Review Board of Konkuk University Medical Center, Seoul, Korea.

### Depletion of abundant proteins in the AH and fractionation of the AH proteome

Abundant proteins in the AH (e.g., albumin and immunoglobulin G [IgG]) were depleted with Pierce™ Top 12 Abundant Protein Depletion Spin Columns (catalog number: 85165; Thermo Scientific); these proteins included α1-acid glycoprotein, fibrinogen, α1-antitrypsin, haptoglobin, α2-macroglubulin, IgA, albumin IgG, apolipoprotein A-I, IgM, apolipoprotein A-II, and transferrin. From each AH sample, a 90-μL aliquot was applied to a depletion spin column and processed according to the manufacturer’s protocol. Both the flow-through and eluent were subjected to SDS-PAGE, and the protein bands were then excised, sliced into pieces and digested via an in-gel digestion method.

### Fractionation and AH protein digestion

**Fig. 2:**
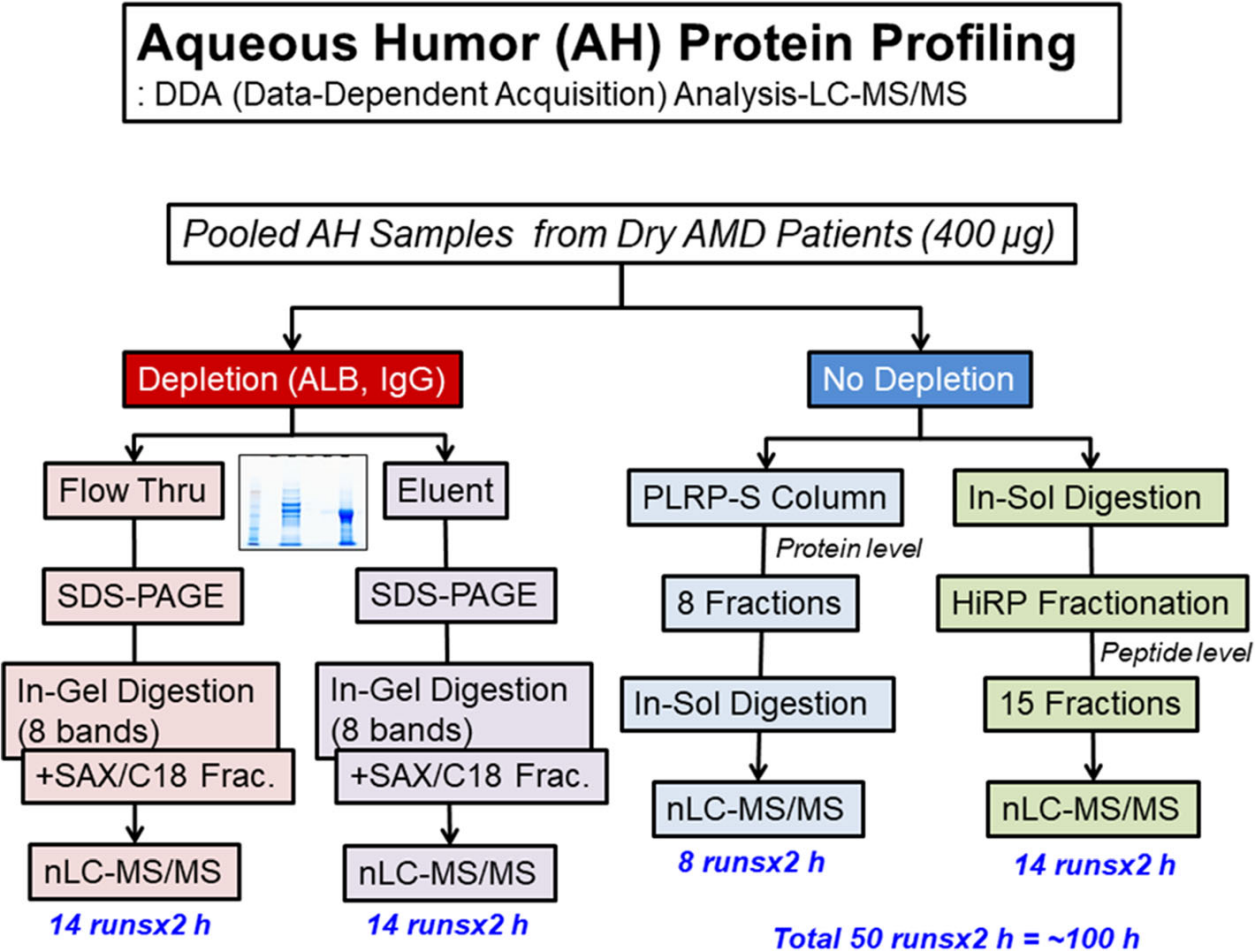
Flowchart of the AH proteome analysis. Pooled AH samples were further prepared via two processes (with or without depletion). A portion of the sample was separated with an ALB/IgG depletion column, and the fractions (flow-through and eluent) were subjected to SDS-PAGE and in-gel digestion (8 bands), followed by multistep fractionation methods (with a strong-anion exchanger and C18 reversed-phase fractionation) at the peptide level. The other portion was subjected directly to in-solution digestion or PLRP-S column chromatography (8 fractions) at the protein level. In-solution-digested samples were further subjected to the fractionation method of high-pH reverse fractionation (15 fractions) at the peptide level, and each PLRP-S fraction was individually digested with trypsin in solution. A total of 50 LC-MS/MS runs were completed, and the total LC-MS/MS running time was more than 100 h

To generate a comprehensive AH proteome library for SWATH-MS analysis, AH samples were processed in multiple ways (Fig. 2): with or without depletion; with in-solution digestion or in-gel digestion; and with protein- (PLRP-S column) or peptide-level fractionation (high-pH fractionation). All proteins obtained following the depletion process (each eluent and flow-through fraction) in SDS-PAGE gels were divided into 8 gel bands and digested into peptides via an in-gel digestion method. In-gel digestion was performed as previously reported [20]. Additionally, for the top 12 abundant protein-depleted samples (7 AMD patients), further fractionation steps were carried out to reduce proteome complexity (14 strong cation exchange fractionation steps and C18 desalting steps). Alternatively, the pooled AH samples that did not undergo depletion were separated with PLRP-S resin, and each fraction was digested through in-solution digestion methods after drying completely. In-solution digestion was performed as previously reported [21]. The AH protein samples were also digested via in-solution digestion methods without any fractionation. In addition, all peptides were fractionated with a DK-Tip C18 HiRP Fractionation kit (Diatech Korea co. Ltd., Seoul, Korea), as per the manufacturer’s manual. Briefly, dried peptide samples were resuspended in 100 mM ammonium formate buffer (pH 10), loaded into an equilibrated DK-Tip C18 HiRP tip with 50 mM ammonium formate buffer (pH 10), washed with 50 μL of 50 mM ammonium formate buffer (pH 10), and then serially eluted with 50 μL of 50 mM ammonium formate buffer with acetonitrile solvent (0, 6, 12, 16, 18, 24, 30, 60%).

### Data-dependent acquisition for protein identification and SWATH-MS

A Triple-TOF™ 5600+ (AB Sciex, Concord, Canada) instrument was utilized for all the experiments as described previously [22]. Briefly, the instrument was coupled with an Eksigent NanoLC-2D+ with nanoFlex cHiPLC system (0.075 mm × 15 cm column) for identification and quantification. Solvent A was composed of 0.1% formic acid/water (*v*/v), and solvent B was composed of 0.1% formic acid/100% acetonitrile (*v*/v). Peptide samples were separated in an analytical column with a linear gradient of 2% solvent B to 35% solvent B over 30 min at a flow rate of 400 nL/min. The Chip nano-LC column was regenerated by washing with 60% solvent B for 50 min and re-equilibrated with 2% solvent B for 10 min. For the data-dependent acquisition (DDA) experiment, a Triple-TOF™ 5600+ mass spectrometer was used at 250 ms for survey scans (TOF-MS) and at 150 ms for automated MS/MS scans for the top 20 ions. The MS/MS triggering criteria for parent ions were as follows: precursor intensity> 135 counts, charge state> 1, with the dynamic exclusion option (exclusion time: 15 s). For SWATH-MS-based experiments, the Triple-TOF™ 5600+ mass spectrometer was used in looped product ion mode, with 20 Da/mass windows (each SWATH window exhibited a 1 Da overlap) in the range of 400 to 1000 Da: Experiment 1: MS1 scan (400~ 1600 Da); Experiment 2: 400~ 420 Da; Experiment 3: 419~ 440 Da… Experiment 31: 979~ 1000 Da. The collision energy for each window was determined based on the appropriate collision energy for a two-charged ion centered on the window, with a spread of 15 eV. An accumulation time of 100 ms was used for each fragment ion scan and for the survey scans (total duty cycle of 3.1 s in high-sensitivity mode).

### Database searching

All spectra generated from the DDA experiment were searched with the MS-GF+ (University of California, San Diego, USA; version Beta v9872) searching algorithm against a simple modified version of the UniProt human protein sequence database (UP000005640_9606_cRAP_AbSeq.fasta: a total of 429,526 protein entries including various antibody sequences and commonly contaminated proteins) with the following search parameters: full tryptic digestion, precursor ion tolerance< 50 ppm, fragment ion mass tolerance< 0.5 Da, fixed modifications for cysteine (+ 57 Da for carbamidomethylation) and biological modifications/artifacts such as methionine oxidation (+ 16 Da).

### Criteria for protein identification

Scaffold (version 4.4.6, Proteome Software Inc., Portland, OR) was used to validate the MS/MS-based peptide and protein identifications. The peptide identifications were accepted if they could be established at greater than 82.0% probability to achieve a false discovery rate (FDR) of less than 1.0% with the Scaffold Local FDR algorithm. Protein identifications were accepted if they could be established at greater 5.0% probability to achieve an FDR of less than 1.0% and contained at least 2 identified peptides. Protein probabilities were assigned by the Protein Prophet algorithm [23]. Proteins that contained similar peptides and could not be differentiated based on MS/MS analysis alone were grouped to satisfy the principles of parsimony. Proteins sharing significant peptide evidence were grouped into clusters. Proteins were annotated with GO (Gene ontology) terms from gene_association.goa_human (downloaded 2014/1/28) [24].

### Proteome data analysis

For SWATH-MS data, all raw data (wiff files) were converted by ProteoWizard software (Version 3.0.6965) into the mz5 format. Transition settings were as follows: MS1 filtering was performed for three isotope peaks in centroid mode with 30 ppm accuracy; MS/MS filtering for three isotope peaks was performed in centroid mode with 50 ppm accuracy. The retention time window was considered to be within 10 min of the MS/MS identification period. Extracted data were manually confirmed considering the retention time and rank order of peak intensities from both the library spectrum and the SWATH spectrum. Perseus™ software was used for further data processing, visualization, principal component analysis (PCA), and other statistical analyses.

## Results

### In vitro primary hfRPE cell culture model under oxidative stress

**Fig. 3:**
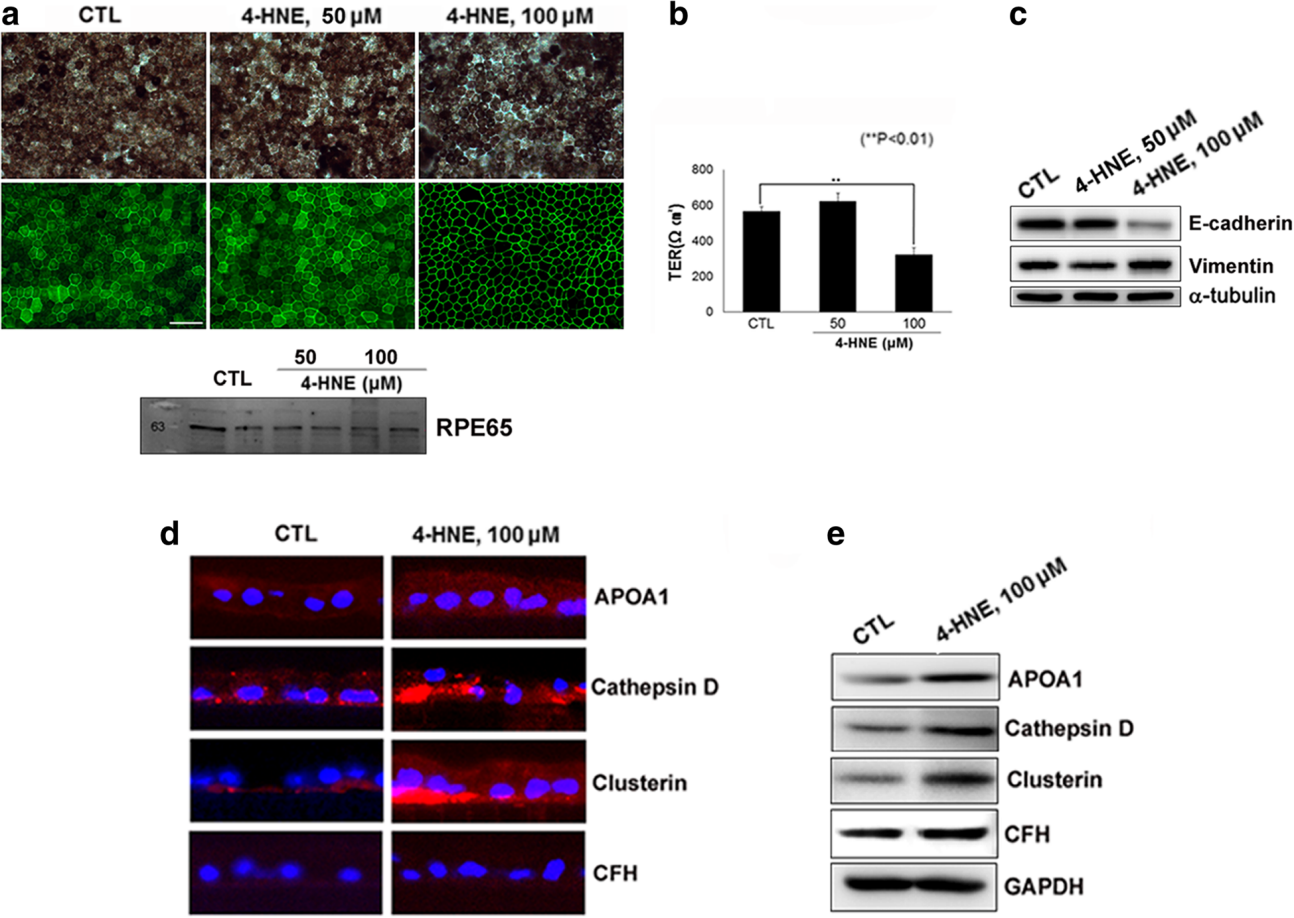
Polarized primary human fetal RPE (hfRPE) cell culture model. Cells were exposed to 4-hydroxy-2-nonenal (4-HNE) for 24 h. (**a**; upper) Phase-contrast microphotographs and microphotographs of the immunofluorescence (IF) staining of hfRPE cultures. (**a**; lower) The expression of RPE65, a RPE-specific protein, was confirmed through Western blot analysis of the lysate of hfRPE cells. (**b**) Quantification of transepithelial resistance (TER). (**c**) Expression of the epithelial marker E-cadherin and the mesenchymal marker vimentin in hfRPE cells exposed to 4-HNE. (**d**) Immunostaining of drusen-related proteins, APOA1, cathepsin D, clusterin and CFH in hfRPE cells. (**e**) Western blot analysis showing increased expression of APOA1, cathepsin D, and clusterin in 100 μM 4-HNE-treated hfRPE cells compared to that in controls

We established a primary hfRPE cell culture model exposed to 4-HNE, one of the major oxidants generated via lipid peroxidation in the retina [25, 26]. This model mimics the in vivo environment of the RPE in patients with dry AMD, although other major oxidation products in the retina are related to the pathogenesis of AMD (Fig. 3).

To evaluate whether the hfRPE cell culture system can mimic and represent the clinical pathologic environment of the RPE in AMD, showing differential apical and basal protein secretion upon oxidative stress, we established an hfRPE Transwell culture system and evaluated monolayer formation by hexagonal, highly pigmented RPE cells and measured their TER. As previously described [18, 27], porous membrane inserts in Transwell cultures generate two compartments: the apical chambers correspond to the retinal-facing side of the RPE (RPD exist in this upper compartment), and the basal chambers correspond to the choroidal-facing side of the RPE (drusen exist in this lower compartment). Monolayers of RPE cells on Transwell plates were treated with 1, 5, 25, 50, or 100 μM 4-HNE for 24 h. No cytotoxicity was observed until the 24 h 50 μM 4-HNE treatment (data not shown). The amount of pigmentation and the immunofluorescence of the tight junction protein ZO-1 showed no significant changes between the hfRPE cells treated with 50 μM 4-HNE for 24 h and those treated under control conditions (Fig. 3a). A high level of TER (500–600 Ω·cm^2^) was also maintained in the RPE cell cultures exposed to 50 μM 4-HNE for 24 h (Fig. 3b). Expression of the epithelial marker E-cadherin and the mesenchymal marker vimentin in hfRPE cells exposed to 4-HNE showed that the epithelial characteristics of hfRPE cells were maintained in the RPE cell cultures exposed to 50 μM 4-HNE for 24 h (Fig. 3c). Then, we identified increased intra- and sub-RPE deposits in the RPE cells exposed to 4-HNE compared to the control cells based on the immunoreactivity of APOA1, cathepsin D, and clusterin, all of which are well-established drusen components [27, 28] (Fig. 3d and e). The change in the level of CFH expression was not significant as shown by both IF and Western blotting.

### Construction of the AH spectral library

Aqueous samples were processed in multiple ways, as indicated in the Methods and in Fig. 2. Protein-level separation (SDS-PAGE after depletion or PLRP-S column treatment) showed a slightly greater number of identified proteins than that achieved by peptide-level separation through high-pH reverse-phase separation. All fractionation and depletion procedures resulted in the identification of a total of 1010 proteins (383 protein groups) in a modified UniProt protein sequence database, including various immunoglobulin sequences (protein and peptide FDR < 1%, ≥two peptides per protein). When various immunoglobulins and other contaminant proteins were excluded, 329 AH proteins (260 clusters) and 3776 peptides were identified. The number of identified mass spectra was 41,903 (FDR < 1%). Among the uniquely identified peptides, the number of components in the spectral library constructed with Skyline software was 6818, which included redundant peptides with different charge states, retention times, and translational modifications.

### Repertoire of the in vivo AH proteome from patients

**Fig. 4:**
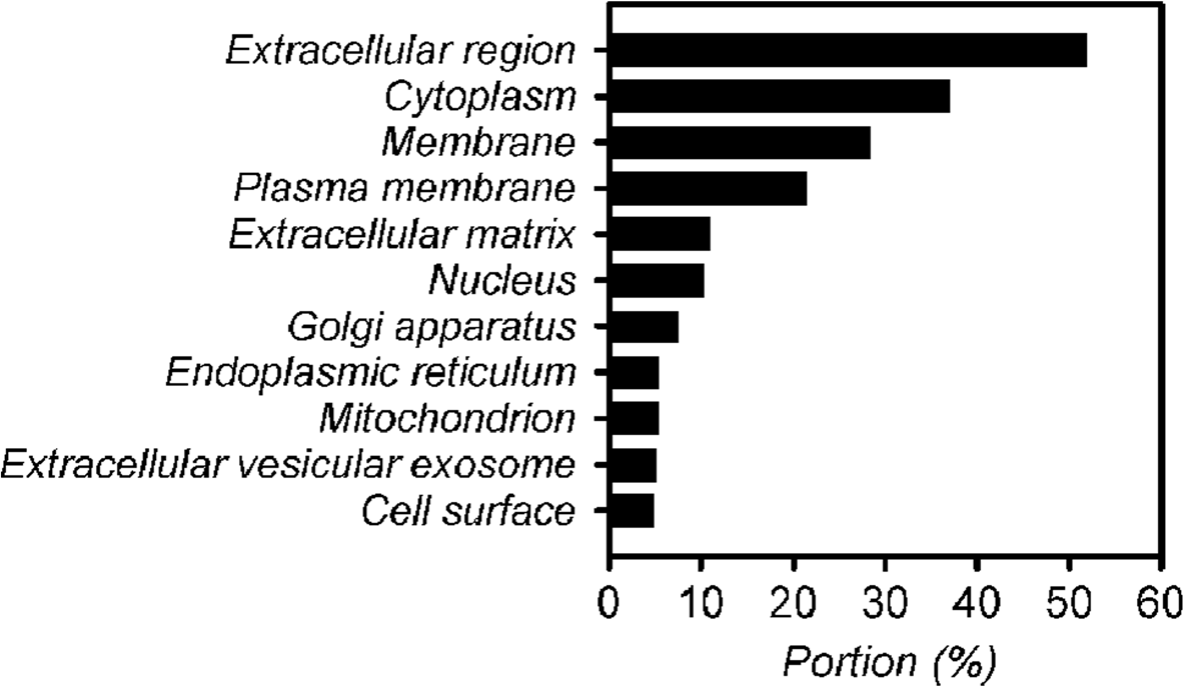
Gene ontology analysis (localization) of the aqueous humor (AH) proteome of dry AMD patients with drusen and reticular pseudodrusen (RPD)

Gene Ontology analysis of the identified AH proteins showed high percentages of extracellular proteins (185 proteins, 51.5%) and membrane proteins (101 proteins, 28.1%) other than the identified antibodies, which were classified as cytoplasmic proteins (36.8%), plasma membrane proteins (21.2%), and extracellular matrix proteins (10.6%). The percentage of all extracellular and membrane proteins reached 65.4% (Fig. 4). The AH proteins contained a high percentage of endogenous antibodies (67.4% of the total proteins). The identified AH proteins included 9 apolipoproteins (APOA1, APOA2, APOA4, APOD, APOE, APOH, APOL1, Clusterin and APOM), 17 complement components (C1q, C1r, C1s, C3, C4A, C5, C6, C7, C8α, C8β, C8γ, C9, D, H, HR1, HR2, and I), 9 proteases, 3 follistatin-related proteins (1, 4, and 5), 5 inter-alpha-trypsin inhibitor heavy chain proteins (H1, H2, H3, H4, and H5), 2 metalloproteinase inhibitors (TIMP1, TIMP2), and 2 retinol-binding proteins (RBP3 and 4). The sequence coverage of RBP3 and RBP4 was 49 and 57%, respectively. Several growth factors were also identified: PEDF, TGFBI, MEGF8, LTBP2, HGFL, HGFAC, GDF8, and 4 insulin-like growth factor-binding proteins (2, 6, 7, and ALS).

### Quantitative analysis of the in vivo AH proteome of patients using SWATH-MS

**Fig. 5:**
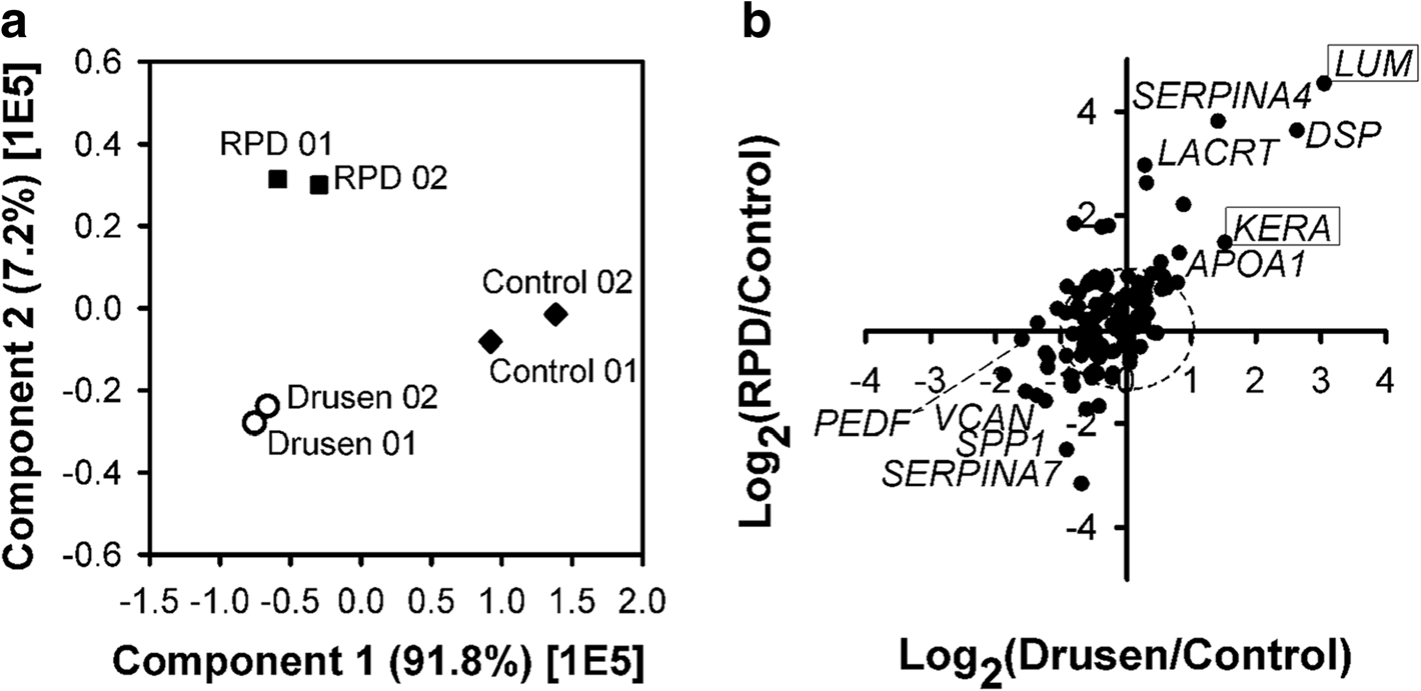
(**a**) Principal component analysis (PCA) of quantified AH proteins from drusen, RPD, and healthy control samples. (**b**) 2D-plot of the relative abundance of AH proteins quantified from drusen and RPD patients versus healthy controls. Upregulated proteins are shown in the upper right quadrant and downregulated proteins in the lower left quadrant for both drusen and RPD samples. The dashed line indicates the boundary of changes in abundance according to the 2-fold criterion. Proteins indicated with boxes are new biomarker candidates in dry AMD patients

Based on the AH spectral library, the abundance of each AH protein was extracted from SWATH-MS data for the control (no AMD), drusen patient, and RPD patient samples. The peak processing data acquired from limited clinical samples resulted in the quantification of 119 of 329 proteins (36.2%, Table S3 in Additional file 2). ANOVA (*p*-value< 0.05) showed that 65 proteins presented significantly altered abundance (54.2% of total proteins) among the three groups (control, drusen and RPD). To identify typical proteins related to pathogenesis, we performed 2D-plot analysis of the abundance ratios of the proteins with statistical significance (drusen versus control and RPD versus control) and then performed PCA with the abundance of all quantified proteins (Fig. 5a). As shown in Fig. 5b, most proteins were not prominently altered (73.3%). Eight proteins exhibited 2-fold higher abundance in both the drusen and RPD patients than in the healthy controls (APOA1, KRT79, FSTL5, LACRT, LUM, DSP, SERPINA4, and KERA). In contrast, VCAN and 18 proteins showed a 2-fold lower abundance in both the drusen and RPD patients (e.g., IGHV3–13, and SPP1). Five proteins showed a 2-fold higher abundance in the RPD group but were decreased in the drusen group (e.g., CPAMD8, ALDH3A1). Fig. 5b shows that the LUM, KERA, and VCAN proteins were differentially expressed in both the RPD and drusen groups. While LUM and KERA were upregulated, VCAN was downregulated. PEDF was downregulated in both the drusen and RPD groups but only showed a statistically significant decrease in the drusen group. Interestingly, few proteins were also observed in outlier areas (the second and fourth quadrants of Fig. 5b), indicating upregulation in the drusen group and downregulation in the RPD group or downregulation in the drusen group and upregulation in the RPD group.

### Comparison of key components secreted from hfRPE cells and AH in the context of AMD pathogenesis

The AH proteins from patients with drusen and RPD were further compared with the samples from the healthy controls in terms of relative abundance (Table 2). For these samples, each protein ratio was shown on a log 2 scale: drusen or RPD against the control in AH samples. The differential expression of APOA1 and clusterin in polarized RPE cells with or without 4-HNE treatment was well correlated with the changes in relative abundance observed in the SWATH-MS analysis shown in Table 2: APOA1 was increased in both the drusen and RPD samples, and clusterin was increased in the RPD sample (Fig. 3d and e).

**Table 2:** Comparison of changes in protein abundance

Gene Name	# of Total Spectral Counts	Fold Change by Quantitative SWATH-MS Analysis		
		AH (Drusen)	AH (RPD)	Comments
APOA1	300	+ 0.82	+ 1.28	Drusen marker
APOA4	149	+ 0.22	−0.53	Lipid metabolism
CFHR2	10	−1.20	−0.92	Lipid metabolism
CTSD	101	0.00	−0.53	Autophagolysosomal
SERPINA4	46	+ 1.42	+ 3.82	Antiangiogenesis
LUM	39	+ 3.05	+ 4.54	Extracellular matrix, Regulation of transcription
KERA	3	+ 1.52	+ 1.48	Extracellular matrix, Keratan (Cornea)
CLUS	93	−0.74	+ 0.25	Clearance of cellular debris
PEDF	407	−1.36	−0.07	Neurotrophic property
PTGDS	239	−0.51	−1.09	Neuromodulator
TIMP1	20	−0.88	−1.06	EMT^a^ marker

^a^*EMT* epithelial-mesenchymal transition

**Fig. 6:**
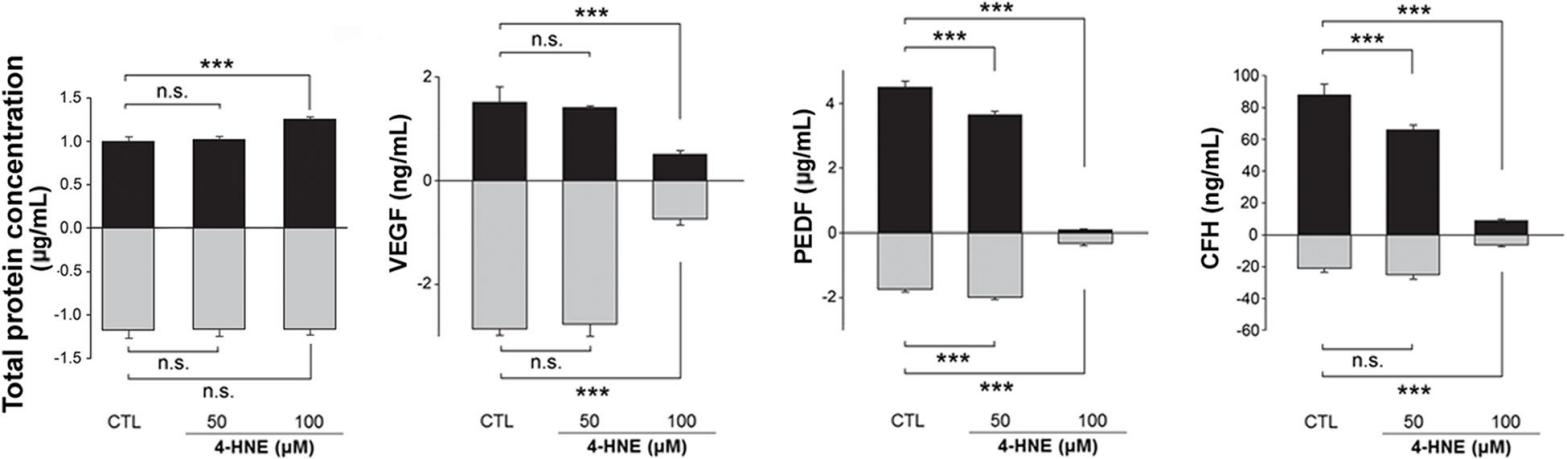
Differential secretion of selected proteins in hfRPE cell cultures. Total protein concentrations in conditioned media from apical and basal baths (left end). To confirm the biological activity of VEGF, PEDF, and CFH, apical or basal media were collected at 24 h and then analyzed using ELISAs (the rest three graphs). The amount of VEGF protein in the basal media was higher than that in the apical media (2.86 ng/mL vs. 1.51 ng/mL) and was greatly decreased by 100 μM 4-HNE treatment (0.74 ng/mL vs. 0.51 ng/mL). PEDF secretion on the apical side was higher than that on the basal side (4.51 μg/mL vs. 1.74 μg/mL), and the level of PEDF was significantly decreased in a dose-dependent manner at 24 h (0.086 μg/mL and 0.32 μg/mL, in apical and basal baths treated with 100 μM 4-HNE). The ratio of the PEDF concentrations secreted in the apical and basal chambers was reversed in cultures treated with 100 μM 4-HNE. The apical/basal ratios were 2.59 and 1.83 in control cultures and cultures exposed to 50 μM 4-HNE for 24 h; the apical/basal ratio was 0.27 in cultures exposed to 100 μM 4-HNE for 24 h. The level of CFH showed similar changes: it decreased both apically and basally in cultures exposed to oxidative stress compared to control cultures. The apical/basal ratios were 4.19 and 2.64 in control cultures and cultures exposed to 50 μM 4-HNE for 24 h, respectively; the apical/basal ratio was 1.41 in cultures exposed to 100 μM 4-HNE for 24 h

We then tested selective proteins from the hfRPE cell culture media and compared the changes in abundance from the differentially expressed protein (DEP) list (RPD versus control: SWATH-MS analysis) with those from the hfRPE culture media (with versus without 4-HNE treatment: ELISA). In control cultures and cultures exposed to 50 μM 4-HNE for 24 h, the total protein concentration in the conditioned media from the basal chamber was higher than that in the media from the apical chamber (the ratios of the apical to basal concentrations were 0.85 and 0.88, respectively), consistent with previous reports that a large majority of proteins are secreted in the basolateral direction [14, 15]. However, in the cultures exposed to 100 μM 4-HNE for 24 h, the total protein concentration in the conditioned media from the apical chamber was higher than that in the media from the basal chamber (the ratio of the apical to basal concentration was 1.08; Fig. 6). In conjunction with the decreases in TER, ZO-1 immunoactivity, and the expression of the epithelial marker E-cadherin in cultures exposed to 100 μM 4-HNE for 24 h (Fig. 3a, b, and c), the difference in the total protein concentration between the apical and basal chambers suggested that polarized epithelial cells might lose their polarity and cell-cell adhesions and acquire the mesenchymal characteristics of motility and invasiveness.

Among the 11 proteins listed in Table 2, the relative abundance of PEDF and CFH was compared between the AH samples from patients and the RPE cell culture media samples (Fig. 6). The level of PEDF decreased both apically and basally in the cultures exposed to oxidative stress compared to that in the control cultures, whereas the ratio of the protein concentrations secreted by the apical and basal chambers was reversed in the cultures treated with 100 μM 4-HNE compared to that in the control cultures. The apical/basal protein concentration ratios were 2.59 and 1.83 in the control cultures and the cultures exposed to 50 μM 4-HNE for 24 h, respectively; the apical/basal ratio was 0.27 in the cultures exposed to 100 μM 4-HNE for 24 h. The level of CFH showed similar changes: it was decreased both apically and basally in the cultures exposed to oxidative stress compared to the level in the control cultures. The apical/basal ratios were 4.19 and 2.64 in the control cultures and the cultures exposed to 50 μM 4-HNE for 24 h, respectively; the apical/basal ratio was 1.41 in the cultures exposed to 100 μM 4-HNE for 24 h.

## Discussion

In the current study, we first investigated the AH proteome of dry AMD patients using a data-independent acquisition method (SWATH-MS), according to patient phenotype. We introduced an hfRPE culture model under oxidative stress that mimics an abnormal proteomic status reflecting AMD and used conventional methods (e.g., ELISA, IF, and WB) to confirm that a set of disease-associated candidate proteins was differentially expressed in polarized hfRPE cell cultures.

### Drusen-related pathogenic environment in dry AMD mimicked by a polarized RPE cell culture model and quantitative proteomics

In the current study, we introduced an hfRPE cell culture model with a proteome that resembles the proteome of the AH from patients with drusen and RPD. Eight of eleven proteins with differential abundance between the controls and dry AMD patients in SWATH-MS analysis (Table 2) were previously considered to be major components or regulators in drusen [27, 28]. Among these proteins, APOA1, CTSD, and CLUS were found to be elevated via IF and WB analysis in the RPE cell cultures treated with 4-HNE compared to the levels in the control cultures. In all the patient AH samples, various immunoglobulins were identified, which accounted for one-third of the total protein abundance (based on spectral counts). This high abundance of endogenous immunoglobulins detected in AH has also been observed in other body fluids (e.g., blood), suggesting that immunologic dysregulation is one of the major pathogenic mechanisms of dry AMD. Complement factor H, one of the most important soluble (secretory) complement regulatory proteins and a major inhibitor of alternative pathways providing protection against harmful complement activation [29], was decreased in both the AH from patients with drusen and RPD and the conditioned media from both apical and basal baths of cells exposed to oxidative stress in the present study. In a previous histologic study using AMD donor eyeballs, CFH immunoreactivity was identified in drusen, the sub-RPE space, along Bruch’s membrane, and in the walls of choriocapillaris [30]. While some authors have reported decreased CFH expression in Bruch’s membrane/choroid complexes in cases of both early AMD and geographic atrophy [31], others have found no difference in the amount of CFH proteins in Bruch’s membrane/choroid tissue samples from AMD patients and controls [32]. Reduced expression of CFH has been observed in senescent or oxidative stressed RPE cell lines (ARPE-19 cells) [33, 34] suggesting that degenerative RPE cells might not be capable of synthesizing complement regulators for self-preservation under conditions of a dysregulated complement pathway; however, the changes in the intracellular expression level of CFH have not been described in RPE from donor eyeballs or polarized hfRPE cultures. Thus, we speculate that secreted CFH, regardless of its intracellular expression level in RPE, decreases during the progression of AMD, leading to the accumulation sub-RPE deposits [35]. Various oxidation products, including advanced glycation end products such as carboxymethyllysine and reactive aldehydes such as carboxyethylpyrrole, malondialdehyde, and 4-HNE, have been shown to accumulate in the retina and cause damage to the retina during the progression of degeneration, as observed during AMD [36–38]; thus, all the phenotypes and pathologies of the RPE observed in vivo cannot be recapitulated by the application of any single agent to a culture system in vitro. However, we were able to demonstrate the compensatory role of our in vitro culture system, which reflects proteomics data from dry AMD patients, in the present study.

The RPE cell is a highly polarized cell types that produces and secretes proteins onto at basolateral and apical surface, meeting differential demands on either side of the photoreceptors and choriocapillaris and holding a large majority of secreted proteins on the basolateral side [14, 15]. In the RPE cultures exposed to oxidative stress in the present study, however, the ratios of secreted proteins between chambers corresponding to the basal and apical surfaces were reversed; the total protein concentrations in the conditioned media from the apical and basal chambers in the in vitro culture model exposed to oxidative stress were different (apical>basal) from those in the control cultures (basal>apical). Likewise, each AH sample (control, drusen, and RPD) exhibited a different total protein concentration (RPD > drusen>control). Epithelial-mesenchymal transition (EMT) is a complicated phenomenon through which polarized epithelial cells lose their polarity and cell-cell adhesions and acquire the mesenchymal characteristics of motility and invasiveness [39, 40]. Thus, it is plausible that a dysfunctional or stressed RPE cell may lose its polarity and increasingly misdirectionally secrete proteins onto the apical surface rather than onto the basolateral surface, resulting in accumulation in the subretinal space (forming RPD) rather than in the sub-RPE space. We speculate that different locations or subtypes of material deposition are associated with different statuses of the RPE, especially the maintenance of proper polarity. In terms of their composition, drusen and RPD have been shown to share several common components, including membranous vesicles, vitronectin, CFH, and apolipoproteins [6, 41–43]; however, their lipid composition is distinct, with a higher concentration of unesterified cholesterol being found in RPD [44–46].

### New candidate drusen markers

Among the 11 proteins from the AH of dry AMD patients listed in Table 2, SERPINA4, LUM, and KERA have not been described among the components of drusen and have not been previously related to dry AMD, although SERPINA4 and LUM have been reported to be elevated in the retina in an ultraviolet (UV)-induced rat model of AMD [47]. The expression level of SERPINA4 in patients with drusen was also higher than that in healthy controls. One of the serine protease inhibitors, SERPINA4-like PEDF (Synonym: SERPINF1), plays a role as a potent negative regulator of angiogenesis. Interestingly, the LUM and KERA proteins were upregulated in both RPD and drusen. These two proteins are well-known for their roles in keratan sulfate proteoglycan (PG) biosynthesis in the cornea [48]. KERA has also been reported as a noncorneal keratan sulfate, such as fibromodulin, that contributes to retinal damage and repair [49] and as sulfated lactosamine, which is a common component of cell surface and extracellular glycoproteins [49]. A small amount of KERA has been found to exist in many other tissues, including the brain [50], thus suggesting its potential active role in the cellular processes of tissues. The known molecular functions of LUM or biological processes related to LUM include the regulation of transcription from the polymerase II promoter [47], collagen binding, extracellular matrix organization and the inflammatory response [51]. LUM has also been identified as a biomarker of UV toxicity associated with disruption of the interphotoreceptor matrix, indicating a role of LUM in photoreceptor cell dysfunction [47].

Although the precise role of PGs in AMD has not yet been fully investigated, the roles of heparan sulfate PG (HSPG) and chondroitin sulfate PG (CSPG) in the thickening of Bruch’s membrane in early AMD have been demonstrated [52]. Roles of PGs in complement regulation have also been suggested; for example, HSPG in Bruch’s membrane provides binding sites for CFH [53]. Further research is needed to explore the specific roles of lumican in the development and progression of AMD because lumican is found in RPE microvilli in addition to the cornea [54]. Thus, we propose these three proteins as new biomarkers for drusen.

## Conclusions

In conclusion, the detailed proteomic analysis of dry AMD patients using SWATH-MS conducted in this study suggests a possible molecular link between in vivo disease processes and different AMD phenotypes and thus provides insights into the in vivo biology of drusen and RPD.

## Additional files

Additional file 1: **Tables S1 and S2.** List of antibodies used for immunostaining and Western blotting. (DOCX 17 kb)

Additional file 2: **Table S3.** Quantitative results of 119 aqueous humor proteins from control, drusen, and reticular pseudodrusen (RPD) samples. The area of all 119 aqueous humor proteins and the ratios of each protein area between control versus drusen and control versus RPD were included. (XLSX 31 kb)

## Abbreviations

AH: aqueous humor
AMD: age-related macular degeneration
BCA: bicinchoninic acid
BM: Bruch’s membrane
CFH: complement factor H
CNV: choroidal neovascularization
CSPG: chondroitin sulfate PG
EMT: epithelial-mesenchymal transition
GA: geographic atrophy
hfRPE: human fetal RPE
HSPG: heparan sulfate PG
IF: Immunofluorescence
PFA: paraformaldehyde
PG: proteoglycan
PVDF: polyvinylidene difluoride
RIPA: radioimmunoprecipitation assay
RPD: reticular pseudodrusen
RPE: retinal pigment epithelium
SWATH-MS: sequential window acquisition of all theoretical fragment ion mass spectrometry
TER: transepithelial resistance
TIMP3: tissue inhibitor of metalloproteinase-3
UV: ultraviolet
VEGF: vascular endothelial growth factor
