# Supplementary material for: Quantitative proteomic analysis of aqueous humor from patients with drusen and reticular pseudodrusen in age-related macular degeneration
Source: BMC Ophthalmol. 2018 Nov 7;18:289. doi: 10.1186/s12886-018-0941-9 (PMC6222993; doi:10.1186/s12886-018-0941-9)
Supplement: Supplementary file 1 — Tables S1 and S2. List of antibodies used for immunostaining and Western blotting. (DOCX 17 kb) [file 12886_2018_941_MOESM1_ESM.docx]

**Table S1.** List of antibodies used for immunostaining.

| Antigen | Species | Dilution | Source |
| --- | --- | --- | --- |
| APOA1 | Rabbit | 1:500 | Santa Cruz |
| Cathepsin D | Rabbit | 1:500 | Santa Cruz |
| Clusterin | Rabbit | 1:250 | Santa Cruz |
| CFH | Mouse | 1:100 | Santa Cruz |
| Alexa Fluor 555 | Rabbit | 1:250 | Life Technologies |
| Alexa Fluor 555 | Mouse | 1:250 | Life Technologies |

**Table S2.** List of antibodies used for Western blotting.

| Antigen | Species | Dilution | Source |
| --- | --- | --- | --- |
| APOA1 | Rabbit | 1:2000 | Santa Cruz |
| Cathepsin D | Rabbit | 1:2500 | Santa Cruz |
| Clusterin | Rabbit | 1:2000 | Santa Cruz |
| CFH | Mouse | 1:1000 | Santa Cruz |
| GAPDH | Mouse | 1:2500 | Abcam |
| Anti-Mouse HRP, IgG antibody | Mouse | 1:2500 | Cell signaling |
| Anti-Rabbit HRP, IgG antibody | Rabbit | 1:2500 | Cell signaling |
